# A novel NEDD4L-TXNIP-CHOP axis in the pathogenesis of nonalcoholic steatohepatitis

**DOI:** 10.7150/thno.81192

**Published:** 2023-04-09

**Authors:** Qian Guo, Mingyang Xin, Qingchun Lu, Dechun Feng, Vicky Yang, Lee F. Peng, Kelly A. Whelan, Wenhui Hu, Sheng Wu, Xiaofeng Yang, Hong Wang, Brad S. Rothberg, Ana M. Gamero, Glenn S. Gerhard, Bin Gao, Ling Yang

**Affiliations:** 1 Department of Medical Genetics and Molecular Biochemistry, Lewis Katz School of Medicine at Temple University, Philadelphia, Pennsylvania, USA.; 2 Laboratory of Liver Diseases, National Institute on Alcohol Abuse and Alcoholism, National Institutes of Health, Bethesda, Maryland, USA.; 3 Division of Hepatology, Lewis Katz School of Medicine at Temple University, Philadelphia, Pennsylvania, USA.; 4 Department of Cancer and Cellular Biology, Lewis Katz School of Medicine at Temple University, Philadelphia, Pennsylvania, USA.; 5 Department of Cardiovascular Sciences/Center for Metabolic Disease Research, Lewis Katz School of Medicine at Temple University, Philadelphia, Pennsylvania, USA.

**Keywords:** TXNIP, CHOP, E3 ligase, Ubiquitination, NASH

## Abstract

**Background:** Nonalcoholic steatohepatitis (NASH) is a leading cause of chronic liver diseases worldwide. There is a pressing clinical need to identify potential therapeutic targets for NASH treatment. Thioredoxin interacting protein (*Txnip*) is a stress responsive gene that has been implicated in the pathogenesis of NASH, but its exact role is not fully understood. Here, we investigated the liver- and gene-specific role of *Txnip* and its upstream/downstream signaling in the pathogenesis of NASH.

**Methods and Results:** Using four independent NASH mouse models, we found that TXNIP protein abnormally accumulated in NASH mouse livers. A decrease in E3 ubiquitin ligase NEDD4L resulted in impaired TXNIP ubiquitination and its accumulation in the liver. TXNIP protein levels were positively correlated with that of CHOP, a major regulator of ER stress-mediated apoptosis, in NASH mouse liver. Moreover, gain- and loss-of-function studies showed that TXNIP increased protein not mRNA levels of *Chop* both *in vitro* and *in vivo*. Mechanistically, the C-terminus of TXNIP associated with the N-terminus of the α-helix domain of CHOP and decreased CHOP ubiquitination, thus increasing the stability of CHOP protein. Lastly, selective knockdown of *Txnip* by adenovirus-mediated shRNA (not targets *Txnip* antisense lncRNA) delivery in the livers of both young and aged NASH mice suppressed the expression of CHOP and its downstream apoptotic pathway, and ameliorated NASH by reducing hepatic apoptosis, inflammation, and fibrosis.

**Conclusions:** Our study revealed a pathogenic role of hepatic TXNIP in NASH and identified a novel NEDD4L-TXNIP-CHOP axis in the pathogenesis of NASH.

## Introduction

Non-alcoholic fatty liver disease (NAFLD) has emerged as the most common cause of chronic liver disease. It is also a hepatic manifestation of the metabolic syndrome associated with insulin resistance and related disorders including obesity, dyslipidemia, type 2 diabetes mellitus and hypertension 1, 2. The clinicopathological spectrum of NAFLD ranges from simple hepatic steatosis or non-alcoholic fatty liver (NAFL), characterized by the accumulation of triglycerides in hepatocytes, to non-alcoholic steatohepatitis (NASH), which consists of fatty liver with hepatic apoptosis, inflammation, and fibrosis 2-5. Without effective intervention, NASH can eventually progress to end-stage liver diseases such as cirrhosis and hepatocellular carcinoma 2, 3. The “double-hit” theory was considered to contribute to the pathogenesis of NAFLD previously. The “first hit” is mainly the hepatocellular lipid accumulation. The “second hit” has been proposed to include a series of cytotoxic events which lead to liver inflammation. With in-depth study, the “multiple parallel hits” theory has been proposed, which refers to the inter-relationship of insulin resistance, lipotoxicity, oxidative stress, endoplasmic reticulum (ER) stress, and gut-microbiota dysfunction 6-8. However, the molecular pathogenesis of NASH is not fully understood.

Thioredoxin interacting protein (*Txnip*) (also known as *Tbp-2*, VDUP1, or *Hyplip1*) is a stress-induced α-arrestin protein that binds to and inhibits thioredoxin (TXN), thus affecting cellular redox balance 9. Increasing TXNIP impairs the reducing activity of TXN, which leads to increased oxidative stress by generating excess reactive oxygen species (ROS) 10. Therefore, TXNIP has been considered a key mediator of oxidative stress. TXNIP also functions through TXN-independent pathways such as regulating gene expression and translocation of glucose transporters 11-17. TXNIP is an essential metabolic regulator in multiple signaling pathways such as glucose and lipid metabolism, inflammation, and apoptosis 18, 19. For example, TXNIP induces β-cell apoptosis and reduces insulin production in the pancreas 18, 20, and promotes hepatic glucose production and insulin resistance of peripheral tissues 21, 22. Therefore, TXNIP has been considered as a promising target for metabolic disorders, such as diabetes in which TXNIP inhibitor has been tested in clinical trial 18. However, the role of TXNIP in NAFLD remains elusive.

In line with increased oxidative stress, TXNIP protein levels are increased in the liver samples of patients and animal models with NAFLD 23-25. Multiple compounds that ameliorate NAFL and/or NASH, including salidroside, salvianolic acid A, quercetin plus allopurinol, and retinoic acid, function through inhibition of hepatic *Txnip* expression 26-29, suggesting a pathogenic role of TXNIP in NASH development. However, inconsistent results were obtained from whole-body Txnip knockout (KO) mouse studies which reported pathogenic roles 23, 30, 31 and protective roles 24, 32 for TXNIP in NASH. Therefore, the role of TXNIP in NASH is still controversial. The use of whole-body *Txnip* KO is insufficient to tease out tissue-specific gene function. In addition, *Txnip* KO is also unable to tease out gene-specific function since *Txnip* KO also deletes the *Txnip* antisense long non-coding RNA (lncRNA) *Gm15441*, which is transcribed from the opposite strand of the *Txnip* gene and functions in energy metabolism and inflammation 33-35. Therefore, the liver- and gene-specific role of *Txnip* in NASH is not clearly elucidated.

In this study, we found that impaired ubiquitination leads to the accumulation of TXNIP protein in NASH mouse liver. The increased TXNIP protein binds to and inhibits the degradation of CCAAT/enhancer-binding protein homologous protein (CHOP), which is a major transcriptional regulator of ER stress-mediated apoptosis and has been reported to regulate apoptosis, inflammation, and fibrosis in NASH 36-39. *Txnip*-specific knockdown (without affecting its antisense lncRNA *Gm15441*) in mouse liver using adenovirus reduced CHOP protein levels and its downstream targets in the apoptotic pathway, and attenuated hepatic apoptosis, inflammation, and fibrosis. These findings support the pathogenic role of hepatic TXNIP in NASH development and provide a better understanding of the molecular pathogenesis of NASH.

## Materials and Methods

### Animal experiments

All animal protocols were approved by Temple University Institutional Animal Care and Use Committee (IACUC). Male C57BL/6J mice purchased from the Jackson Laboratory were housed in a pathogen-free animal facility at 22 ± 2 °C under controlled 12-h light/dark cycles and randomly assigned to control and experimental groups. Mice were given regular chow (control) or a special diet and had access to autoclaved water *ad libitum*. MCD NASH model: Mice (8 weeks old) were fed a Methionine and Choline Deficient L-Amino Acid Diet (A02082002BR, Research Diets Inc.) for 4 weeks. CDAHFD NASH model: Mice (8 weeks old) were fed an L-Amino Acid Diet With 60 kcal% Fat With 0.1% Methionine and No Added Choline (A06071302, Research Diets Inc.) for 12 weeks. GAN NASH model: Mice (8 weeks old) were fed a GAN Diet With 40 kcal% Fat (Mostly Palm Oil), 20 kcal% Fructose and 2% Cholesterol (D09100310, Research Diets Inc.) for 16 weeks. Age-associated NASH model: Mice (55 weeks old) were fed a GAN diet (D09100310, Research Diets Inc.) for 4 weeks. For Txnip, Chop, or YFP liver-specific overexpression or knockdown, adenovirus was delivered intravenously at 1-2 × 10^9^ plaque-forming units (pfu) per mouse. For GalNAc siRNA-mediated Txnip knockdown, mice were administrated with 2mg/kg GalNAc conjugated control or siTxnip1 through subcutaneous injection. Mice were euthanized after an overnight food withdrawal, and liver and blood samples were harvested for further analysis.

### Isolation of mouse primary hepatocytes and non-parenchymal cells (NPCs)

Immediately after anesthesia with Ketamine (100 mg/kg) and Xylazine (10 mg/kg), mice livers were perfused with Krebs Ringer buffer and digested using collagenase (Liberase TM Research Grade, Roche). Primary hepatocytes (pellet) were collected after centrifugation at 50×g for 5 min. The supernatant was collected to centrifuge at 1500rpm for 10 min to get the NPCs.

### ROS analysis *in vitro*

ROS levels in Hepa1-6 cells were measured using the DCFDA/H2DCFDA-Cellular ROS Assay Kit (ab113851) (Abcam, Cambridge, MA) according to the manufacturer's manual. Briefly, after transfected with siTxnip for 48h, cells were incubated with 20μM DCFDA working solution for 45 min at 37°C in the dark, then further incubated in 1mM H2O2 at 37ºC in the dark for 4 h. DCF intensity was measured on a fluorescence plate reader at Ex/Em = 485/535 nm.

### RNA isolation and RT-PCR

Total RNA was isolated using Trizol reagent (Invitrogen, USA) followed by a Turbo DNase treatment (Ambion) according to the manufacturer's instructions. Next, 0.5 μg RNA was used to perform reverse transcription using SuperScript® III First-Strand Synthesis System (Invitrogen). Real-time polymerase chain reaction amplification of the transcribed cDNA was performed with a real-time PCR system (Mastercycler, Eppendorf). The sequences of the primer sets are listed in Supplementary Table S1. The relative amount of mRNA in each sample was normalized to 18S transcript levels.

### Western blot analysis

Mouse liver samples and cells were disrupted with 1X RIPA lysis buffer (Cell Signaling Technology) supplemented with protease inhibitor and protein phosphatase inhibitor (Thermo Scientific). The protein concentration was quantified using Bio-Rad Protein Assay Dye Reagent Concentrate (Cat. #5000006, Bio-Rad Laboratories, USA). Samples were separated and transferred to polyvinylidene fluoride (PVDF) membranes, then were incubated with their primary antibodies overnight at 4 °C. Primary antibodies used were against TXNIP (1:500, MABS1225, Sigma-Aldrich), CHOP (1:1000, #2895S, Cell Signaling Technology), UBIQUITIN (1:1000, sc-8017, Santa Cruz Biotechnology), BIM (1:1000, #2933T, Cell Signaling Technology), ERO1L (1:1000, 67416-1-Ig, Proteintech), CASPASE 3 (1:1000, 19677-1-AP, Proteintech), CASPASE 7 (1:1000, 27155-1-AP, Proteintech), CASPASE 8 (1:1000, 13423-1-AP, Proteintech), NEDD4L (1:1000, 13690-1-AP, Proteintech), p-AMPK (Thr172) (1:1000, 2535S, Cell Signaling Technology), AMPK (1:1000, 5831S, Cell Signaling Technology), V5 Tag (1:1000, MA5-32053, Invitrogen), 6x-His Tag (1:1000, MA1135, Invitrogen), and β-ACTIN (1:1000, 8457S, Cell Signaling Technology). The following day, membranes were incubated with fluorescence conjugated secondary antibody (LI-COR) for 1 h at room temperature. The protein band was visualized using a quantitative fluorescence imaging system (LI-COR). Band intensities were quantified by using Quantity One software and normalized to β-actin level unless otherwise specified.

### Cell culture, transfection, and treatment

The murine hepatocyte cell lines, Hepa1-6 and AML12, were obtained from ATCC and cultivated in a humidified atmosphere (5% CO_2_, 37°C). Hepa1-6 cells were maintained in Dulbecco's Modified Eagle's Medium (DMEM) (Gibco, 11965-092, USA) supplemented with 10% cosmic calf serum (CCS) (HyClone, SH30087.04, USA) and 1% penicillin streptomycin (Gibco, 15140-122, USA). AML12 cells were grown in a 1: 1 mixture of DMEM (Gibco)/Ham's F-12 with L-glutamine/15 mM HEPES and supplemented with 10% CCS, 1% penicillin streptomycin, 40 ng/ml dexamethasone (Sigma Aldrich), and 1x Insulin-Transferrin-Selenium (Gibco, 41400-045, USA). Lipofectamine 2000 Reagent (Invitrogen, 11668-019) was used for DNA plasmids transfection, and Lipofectamine RNAiMAX Reagent (Invitrogen, 13778-150) was used for small-interfering RNA (siRNA) transfection according to the manufacturer's instructions. The siRNA sequences are listed in Supplementary Table S2.

### Adenovirus production

For overexpression, the coding sequence (CDS) of *Txnip* or *Chop* was obtained by PCR from mouse liver cDNA and subcloned into adenoviral vector pAd/CMV/V5-DEST (Cat.V49320, Invitrogen, USA) according to the manufacturer's protocols. For knockdown, the hairpin template oligonucleotides were synthesized by Integrated DNA Technologies and were subsequently cloned into the adenovirus vector of the pAD/Block-it system (Invitrogen) according to the manufacturer's protocols. The shRNA oligonucleotides are listed in Supplementary Table S3. Adenoviruses were produced and amplified in HEK293A cells. Adenoviruses were purified by CsCl density-gradient ultracentrifugation and desalted using PD10 columns (GE Healthcare Life Sciences). Titration was applied using Adeno-X Rapid Titer Kit (Clontech, USA). Adenoviral transduction of target cells with a level of 50 multiplicity of infection (MOI) was performed to test the expression of target genes.

### Expression Plasmids Constructs

To generate plasmid *Txnip*, *Txnip*-N, *Txnip*-C, *Chop*-6x his, and *Chop* ΔN36, the corresponding complementary DNA (cDNA) fragments were amplified using KOD Xtreme^TM^ Hot Start DNA polymerase (Cat.71975-3, Novagen, USA), then cloned them into the pcDNA^TM^6.2/V5-PL-DEST Mammalian Expression Vector (Cat.12537162, Invitrogen, USA). All of the constructs generated were confirmed by DNA sequencing.

### Immunoprecipitation

Cells were lysed in Lysis Buffer (25 mM Tris-HCl pH 7.5, 150 mM NaCl, 1 mM EDTA, 0.5% Triton X-100, 5% glycerol) added Proteinase Inhibitor Cocktail (Cat.78444, Thermo Scientific, USA) freshly before use. The cell lysates were incubated with anti-CHOP (#2895S, Cell Signaling Technology), anti-TXNIP (#14715S, Cell Signaling Technology), or anti-NEDD4L (13690-1-AP, Proteintech) antibodies overnight. Protein A (Dynabeads^TM^ Protein A, Cat.10002D, Invitrogen) or Protein G (Dynabeads^TM^ Protein G, Cat.10004D, Invitrogen) were pre-cleaned with Lysis Buffer three times and used to precipitate the immune complex. The precipitates were washed with lysis buffer three times, separated by SDS-PAGE, and immunoblotted with indicated antibodies.

### Immunofluorescence

Cells grown on coverslips in a 6-well plate were fixed in 4% paraformaldehyde for 10 min after being rinsed with 1X PBS. Cells were then permeabilized with 0.2% Triton X-100 solution for 5 min. Then, cells were then blocked by 3% BSA (A3294, Sigma-Aldrich) for 1 hour, followed by overnight incubation with anti-CHOP antibody (1:400, #2895S, Cell Signaling Technology) and anti-TXNIP antibody (1:400, #14715S, Cell Signaling Technology) at 4°C. After that, cells were washed three times with 1× PBS and then incubated with the fluorescently labeled secondary antibody (1:400) for 1 hour in the dark at 37°C. Cells were then washed three times (protected from light) and counterstained with nuclear dye DAPI (Cat.D9542, Sigma-Aldrich). The slides were mounted by ProLong^TM^ Diamond Antifade Mountant (Cat.P36965, Invitrogen) and images captured by a Leica Microsystems confocal microscope.

### Liver Histology and Immunohistochemistry

Liver tissues were fixed in 10% formalin, embedded in paraffin, and sectioned at 4 μm. The slides were stained with H&E and Sirius red (Sigma). TUNEL staining was performed according to the manufacturer's instruction of ApopTag Peroxidase *In situ* Apoptosis Detection Kit (S7100, Sigma). Immunohistochemical analysis was conducted by using anti-F4/80 (1:400, #70076, Cell Signaling Technology). The slides were visualized using the Liquid DAB Substrate Chromogen System (Dako). The quantitative analysis of TUNEL, Sirius red, and F4/80 was conducted with Image J or counted.

### Blood and liver metabolite measurements

Blood glucose levels were assayed from a tail-clip using an Ascensia Elite XL glucometer (Bayer Co.). Blood samples were collected after overnight fasting by cardiac puncture using heparinized 25 G needles with 1 mL syringes during terminal anesthesia. Plasma samples were obtained by centrifuging blood samples at 5000×g at 4 °C. Plasma levels of alanine aminotransferase (ALT) were evaluated by ALT (SGPT)-IFCC Liquid Reagent Set (Teco Diagnostics, A524-150). Liver triglyceride (TG) content of mice and plasma levels of TG were assayed by a Triglyceride Determination kit (Sigma, TR0100). Liver total cholesterol (TC) content of mice and plasma levels of TC were assayed by the Total Cholesterol (TC) Colorimetric Assay Kit (Elabscience, E-BC-K109-S).

### Statistical analysis

Results are expressed as the mean ± standard error of the mean (SEM) values. The significance of the difference among all groups was tested by a one-way analysis of variance (ANOVA) test, and the comparison between every two groups was carried out using a student's *t-test*.

## Results

### TXNIP protein accumulates in NASH mouse liver

**Figure 1:**
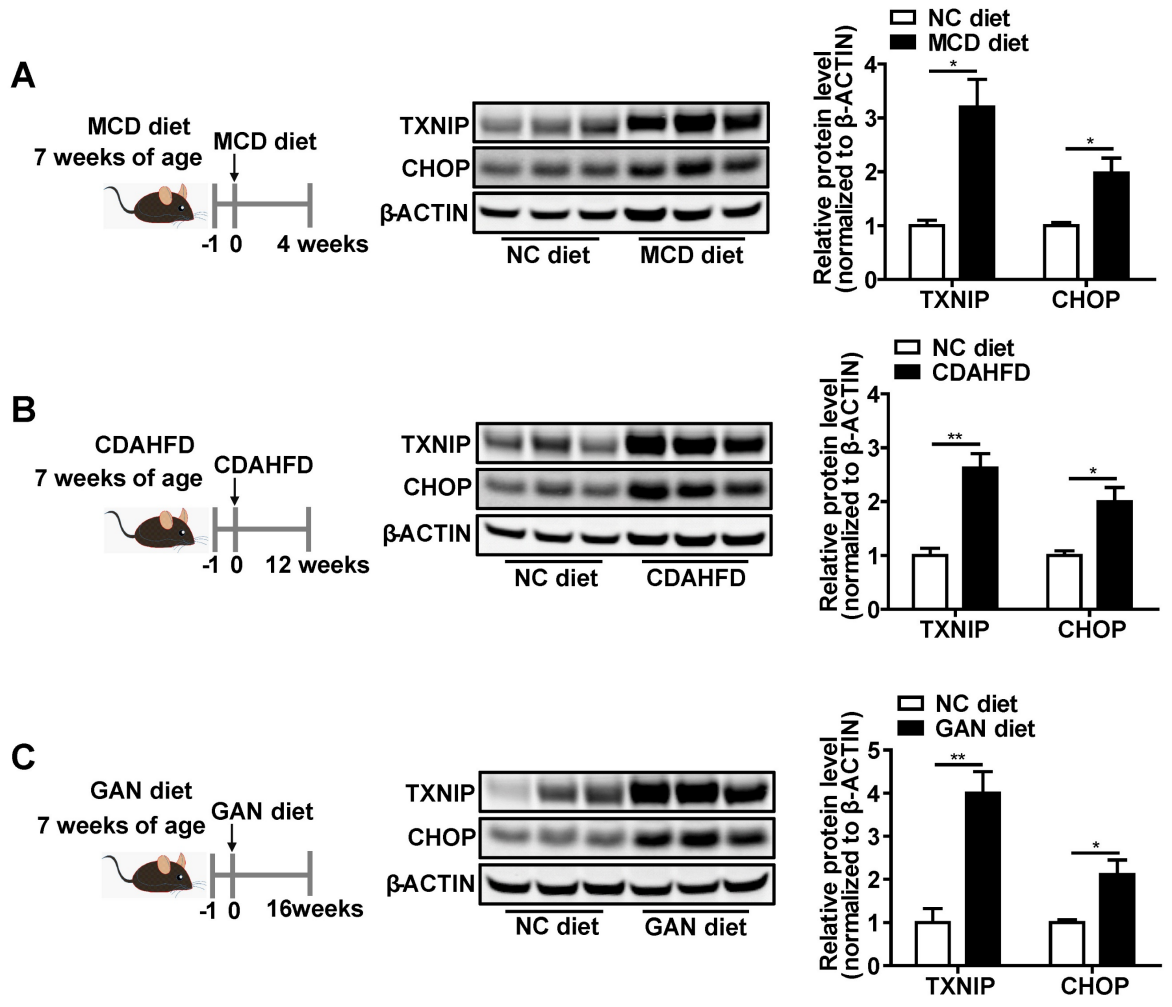
** TXNIP and CHOP proteins accumulate in NASH mouse livers.** (A-C) Schematic diagram of NASH mouse model establishment (Left). Western blot analysis of TXNIP and CHOP in the livers of different NASH mouse models (middle) and its quantification results (right): MCD (4 weeks feeding) NASH mouse model (A), CDAHFD (12 weeks feeding) NASH mouse model (B), GAN (16 weeks feeding) NASH mouse model (C). n = 5. NC, normal chow. Data shown as mean ± SEM (*P < 0.05, **P < 0.01).

Given that the function of *Txnip* in NASH remains elusive, we first measured hepatic expression of TXNIP protein in multiple diet-induced NASH mouse models, including the MCD-, CDAHFD-, and GAN-induced NASH mouse models (Figure 1A-C). Western Blot analysis showed increased protein levels of TXNIP in NASH mouse livers (Figure 1A-C).

**Figure 2:**
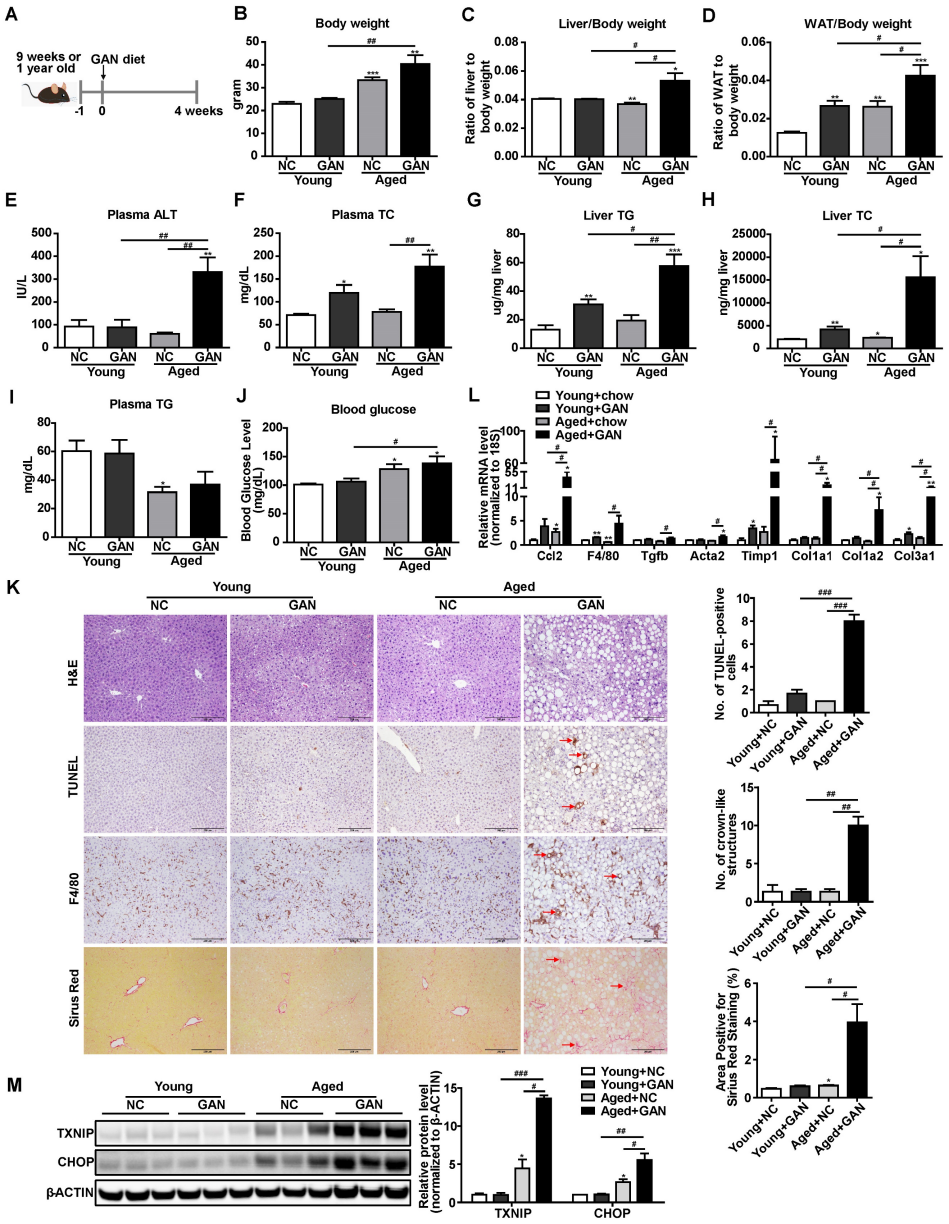
** Hepatic TXNIP and CHOP proteins accumulate in a novel age-associated NASH mouse model.** (A) Schematic diagram of age-associated NASH mouse model establishment (9 weeks or 1-year-old male C57BL/6J mice fed GAN diet for 4 weeks). (B-J) Biochemical analysis of age-associated NASH mouse model. (K) H&E staining, terminal deoxynucleotidyl transferase-mediated dUTP nick end-labeling (TUNEL) staining, immunohistochemical staining for F4/80, and Sirius Red staining of representative liver sections. Scale bar: 200 µm. Red arrows indicate TUNEL-positive cells (in TUNEL staining image) or crown-like structures (in F4/80 staining image) or positive area (in Sirius Red staining image). The quantification results were shown on the right. (L) RT-PCR analysis of inflammation and fibrosis genes in livers of age-associated NASH mouse model. (M) Western blot analysis of TXNIP and CHOP in the liver of age-associated NASH mouse model and its quantification results (right). Data shown as mean ± SEM (n = 4-5. *P < 0.05, **P < 0.01, ***P < 0.001, #P < 0.05, ##P < 0.01, ###P < 0.001, each group compared to the young mice NC group or as indicated).

Aging is a major risk factor for the NAFLD development and an increased susceptibility to develop NASH 40. To take aging into the consideration and further confirm the elevation of TXNIP protein in NASH mouse liver, we also established a novel age-associated NASH model by feeding aged (one-year-old) mice with the GAN diet for 4 weeks (Figure 2A). The body weight, liver weight, white adipose tissue (WAT) weight, plasma alanine aminotransferase (ALT), plasma total cholesterol (TC), liver triglyceride (TG), and liver TC in aged mice fed with GAN diet were significantly increased compared to chow diet-fed aged mice, GAN diet-fed young mice, or chow diet-fed young mice (Figure 2B-H). While no differences were observed between GAN diet and chow diet feeding in plasma TG and blood glucose levels, aged mice with GAN-induced NASH displayed increased blood glucose level as compared to their young counterparts (Figure 2I,J). Hepatic steatosis, TUNEL-positive cells, macrophage infiltration, and fibrosis were also significantly increased in aged mice fed with GAN diet (Figure 2K). In addition, the hepatic expression levels of genes associated with inflammation (*Ccl2* and *F4/80*) and fibrosis (*Tgfb*, *Acta2*, *Timp1*, *Col1a1*, *Col1a2*, and *Col3a1*) were dramatically elevated in aged mice fed GAN diet (Figure 2L). These results indicate that feeding aged mice with GAN diet for 4 weeks can successfully induce NASH. Intriguingly, the protein levels of TXNIP were also accumulated in the livers of this age-associated NASH mouse model (Figure 2M). Taken together, these results robustly demonstrated that TXNIP proteins are accumulated in NASH liver.

### Impaired ubiquitination leads to TXNIP protein accumulation in NASH mouse liver

**Figure 3:**
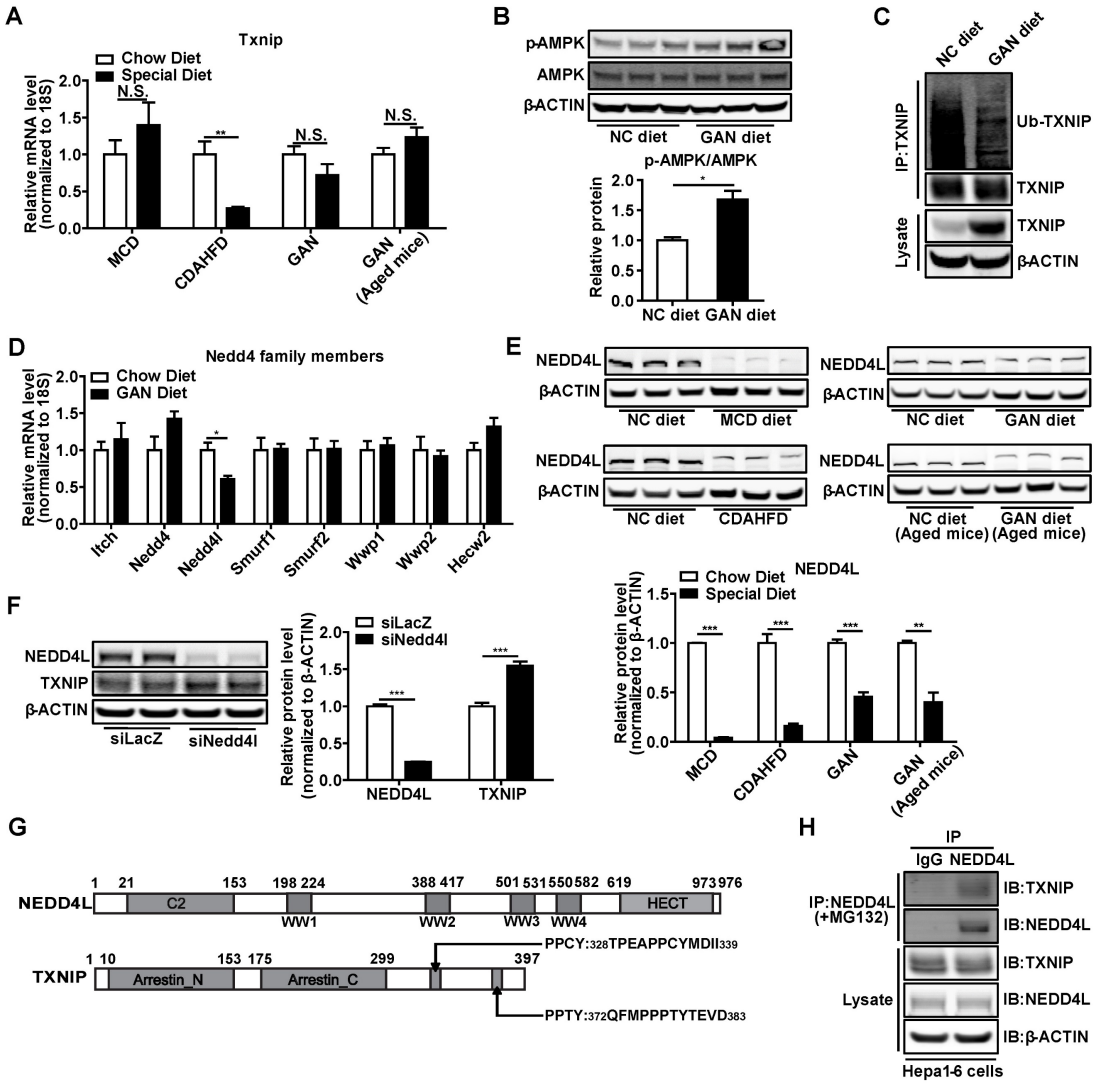
** NASH leads to the accumulation of TXNIP through impaired ubiquitination.** (A) RT-PCR analysis of *Txnip* mRNA levels in the livers of different NASH mouse models. (n = 5). (B) Western blot and quantification analysis of p-AMPK in GAN-NASH mouse model. (C) Ubiquitin-TXNIP (Ub-TXNIP) was determined by immunoprecipitation (IP) of TXNIP with a subsequent Western blot assay with an anti-Ubiquitin antibody in the liver of the GAN-NASH mouse model. (D) RT-PCR analysis of *Nedd4* family members mRNA levels in the liver of GAN-NASH mouse model. (n = 5). (E) Western blot and quantification analysis of NEDD4L in NASH models. (F) Western blot and quantification analysis of TXNIP and NEDD4L in Hepa1-6 cells transfected with siLacZ or siNedd4l for 48 hours. (G) Schematic representation of NEDD4L having 4 WW domains (upper panel) and TXNIP having PPXY motifs (lower panel). (H) Physical interaction between TXNIP and NEDD4L. Hepa1-6 cells were transfected to overexpress *Txnip* for 24 hours and then incubated with 5 μM MG132 for 4 hours. Equal amounts of Hepa1-6 cell lysates were immunoprecipitated with negative control IgG and NEDD4L antibodies and immunoblotted with the indicated antibodies. Data shown as mean ± SEM (*P < 0.05, **P < 0.01, ***P < 0.001, N.S., not significant).

Next, we explored the underlying molecular mechanism through which TXNIP accumulated in NASH mouse liver. *Txnip* mRNA levels were not increased in the livers of NASH mouse models, suggesting that the TXNIP accumulation may be due to post-transcriptional modification (Figure 3A). Recent evidence indicates that phosphorylation of TXNIP by AMP-dependent protein kinase (AMPK) results in TXNIP degradation 11. In NASH mouse livers, western blot analysis revealed increased levels of p-AMPK (Figure 3B), suggesting that TXNIP protein accumulation in NASH is unlikely a result of impaired degradation downstream of APMK. The E3 ubiquitin ligase ITCH has been shown to interact with TXNIP, leading to TXNIP proteasomal degradation 41, 42. Proteins destined for proteasomal degradation are often ubiquitinated. We then determined whether TXNIP underwent ubiquitination in NASH mouse liver. Immunoprecipitation analysis indicated that the ubiquitinated form of TXNIP was dramatically decreased in NASH mouse liver (Figure 3C). These results suggest that the accumulation of TXNIP protein in NASH is due to impaired TXNIP ubiquitination.

To further address the contributions of E3 ubiquitin ligases to accumulation of TXNIP, we assessed gene expression of all the *Nedd4*-like family members, including *Itch* in NASH mice livers. Results showed that *Nedd4l* was dramatically decreased but not *Itch* (Figure 3D). Western Blot analysis also showed that NEDD4L was reduced in the liver of different NASH models (Figure 3E). Knockdown of *Nedd4l* in Hepa1-6 cells significantly increased TXNIP protein levels (Figure 3F). *Nedd4*-like family members contain WW domains that are known to mediate their binding to substrates with the PPXY motifs 43. TXNIP has two PPXY motifs in the C-terminus 42 (Figure 3G) and immunoprecipitation analysis indicated interaction between NEDD4L and TXNIP (Figure 3H). Collectively, these data suggest that deficiency of *Nedd4l* in the context of NASH may limit TXNIP ubiquitination to promote TXNIP accumulation.

### TXNIP leads to CHOP protein accumulation both *in vitro* and *in vivo*

**Figure 4:**
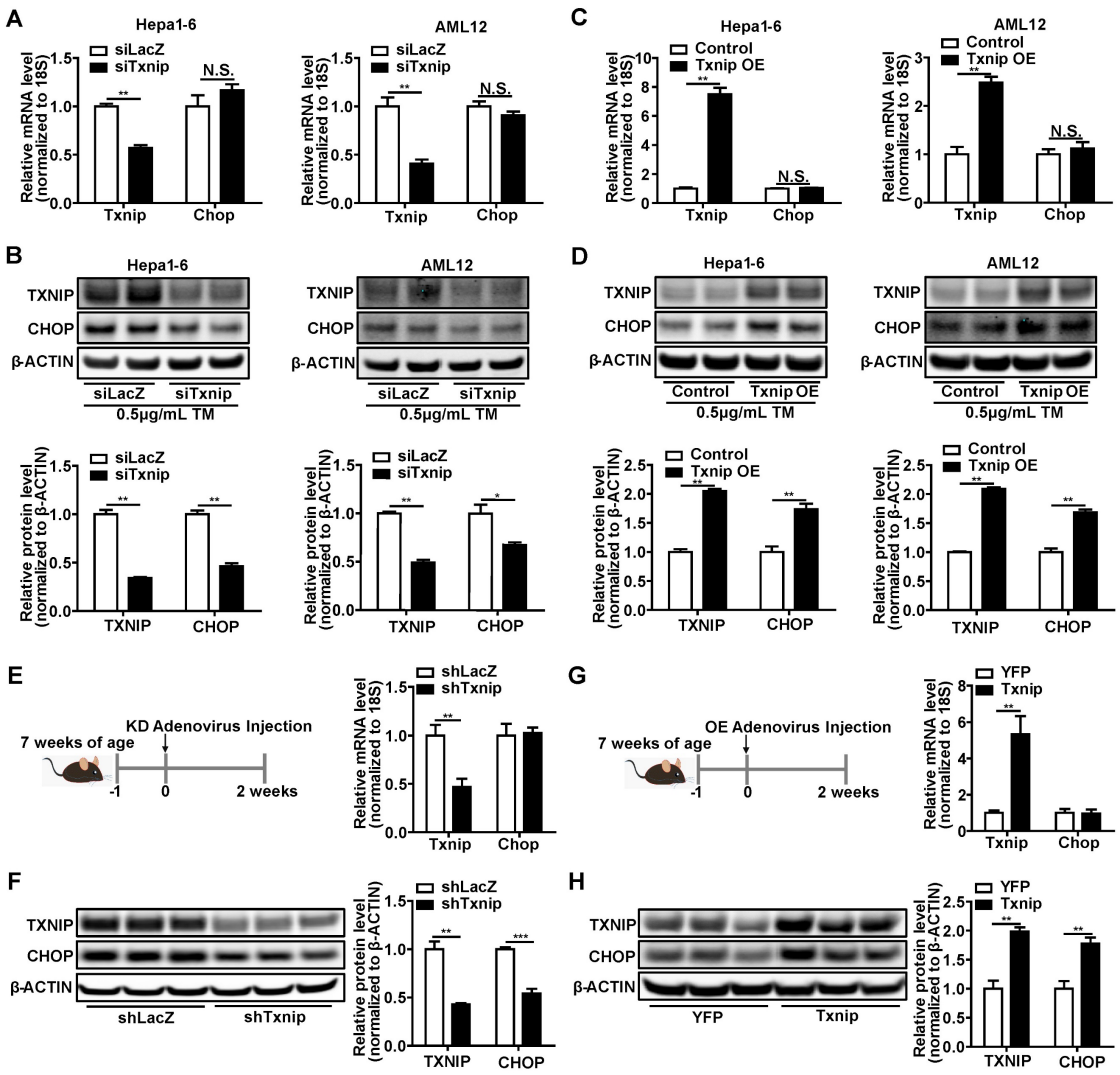
** TXNIP regulates CHOP protein accumulation both *in vitro* and *in vivo*.** (A-B) RT-PCR (n = 4) and Western Blot analysis of *Txnip* and *Chop* in Hepa1-6 and AML12 cells exposed to 0.5 μg/mL Tunicamycin (TM) for 6 hours after transfection with *Txnip* siRNA or control siRNA for 24 hours. (C-D) RT-PCR (n = 4) and Western Blot analysis of *Txnip* and *Chop* in Hepa1-6 and AML12 cells exposed to 0.5 μg /mL Tunicamycin (TM) for 6 hours after transfection with *Txnip* overexpression vector or control vector for 24 hours. (E and F) RT-PCR (n = 5) and Western Blot analysis of *Txnip* and *Chop* in the livers of *Txnip* liver-specific knockdown mice. (G and H) RT-PCR (n = 5) and Western Blot analysis of *Txnip* and *Chop* in the livers of *Txnip* liver-specific overexpression mice. Data shown as mean ± SEM (*P < 0.05, **P < 0.01, ***P < 0.001, N.S., not significant).

CCAAT-enhancer-binding protein homologous protein (*Chop*) is a key transcription factor that plays an essential role in the ER stress-induced apoptosis 36-39. Hepatocyte apoptosis initiates NASH development and leads to inflammation (recruitment of macrophages) and fibrosis (activation of stellate cells) in the liver 44. Due to several CHOP antibodies having been found to be questionable, the CHOP antibody used in this study was first validated in the Supplementary Fig. S1 using ER stress inducer, *Chop* knockdown, and *Chop* overexpression. Intriguingly, we found that the protein levels of CHOP in the liver concurrently increased with TXNIP proteins under NASH conditions (Figure 1A-C and 2M). These results prompt us to determine whether TXNIP regulates CHOP expression. Both *Txnip* loss- and gain-of-function studies were performed in hepatic cell lines and mouse livers. Results from Hepa1-6 and AML12 hepatic cell lines showed that siRNA-mediated *Txnip* knockdown significantly decreased CHOP protein but not mRNA level (Figure 4A,B). *Txnip* overexpression also failed to influence *Chop* mRNA, but significantly increased CHOP protein (Figure 4C,D). In addition, we found that TXNIP regulated cellular ROS levels and ER stress marker proteins such as ATF4, IRE1α, and XBP1 (Supplementary Fig. S2). Consistently, adenovirus-mediated *Txnip* knockdown or overexpression in mouse liver respectively decreased or increased CHOP protein level without affecting *Chop* mRNA level (Figure 4E-H). These data suggest that TXNIP leads to CHOP protein accumulation, both *in vitro* and *in vivo*, which is likely via post-transcriptional regulation.

### TXNIP inhibits ubiquitination-mediated protein degradation of CHOP

**Figure 5:**
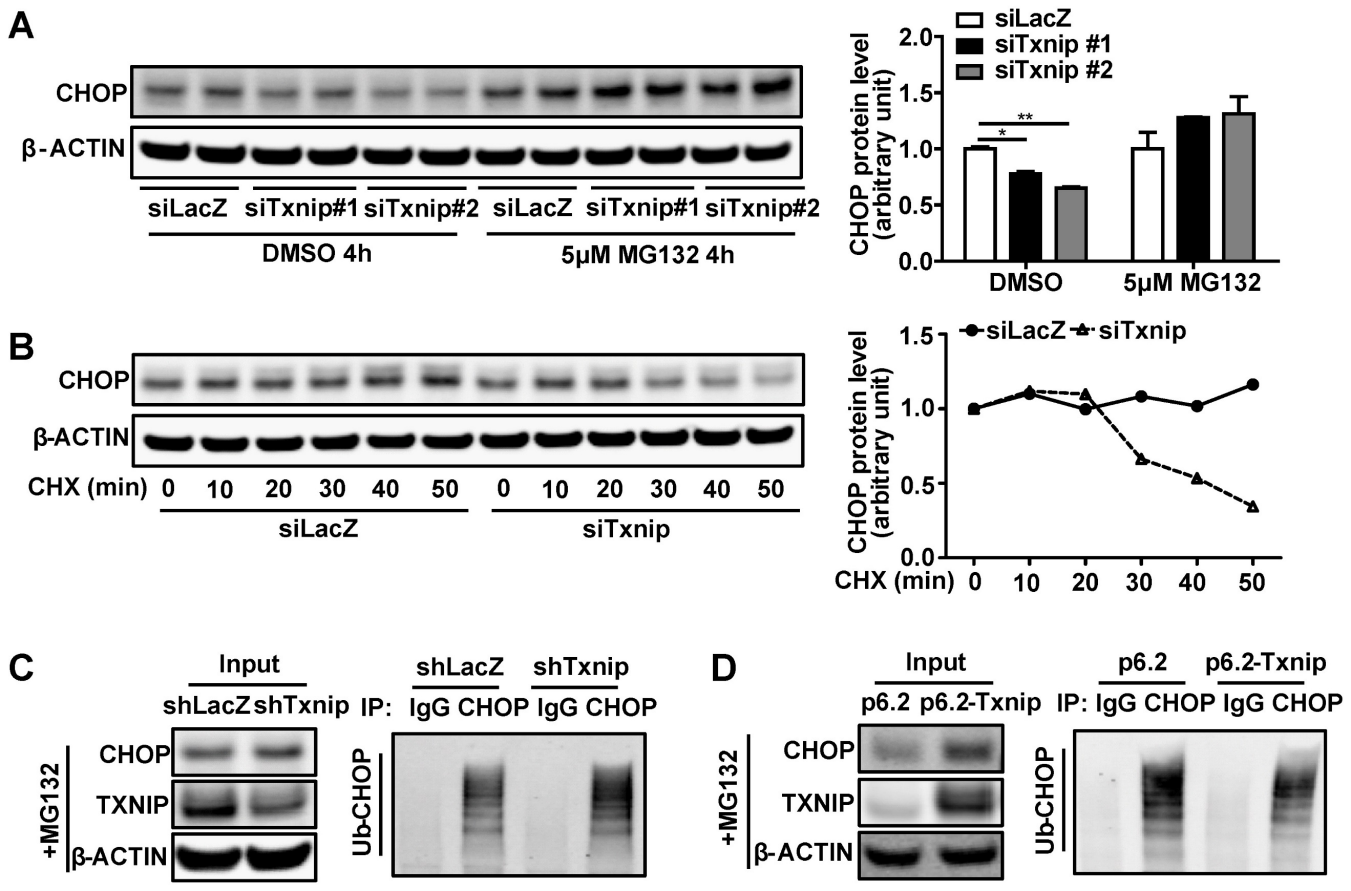
** TXNIP inhibits ubiquitin-proteasome degradation of CHOP.** (A) Representative western blot image of CHOP protein levels (left) and quantification of CHOP band intensities (right) after *Txnip* knockdown in presence or absence of proteasome inhibitor MG132 in Hepa1-6 cells under Tunicamycin treatment. (B) Representative western blot image of CHOP protein levels (left) and quantification of CHOP band intensities (right) in the control and *Txnip* knockdown Hepa1-6 cells in the presence of CHX for the indicated periods under Tunicamycin treatment. (C and D) Ubiquitin-CHOP (Ub-CHOP) was determined by immunoprecipitation (IP) of CHOP followed by western blot in *Txnip* knockdown (C) or overexpression (D) condition. Data shown as mean ± SEM (*P < 0.05, **P < 0.01).

Decreased protein degradation or increased protein synthesis can lead to CHOP protein accumulation without affecting its mRNA level. Therefore, we used the protein degradation inhibitor MG132 and protein synthesis inhibitor cycloheximide (CHX) to determine whether TXNIP regulated CHOP protein degradation or synthesis. We found that *Txnip* KD did not reduce CHOP protein levels when protein degradation was blocked by MG132 (Figure 5A). This result suggests that *Txnip* KD reduces CHOP protein through degradation. In support of this, when protein synthesis was blocked by CHX, *Txnip* KD accelerated the degradation rate of CHOP protein (Figure 5B). These results indicate that TXNIP protects CHOP from protein degradation rather than increasing protein synthesis. Ubiquitination is a vital mechanism that controls CHOP protein degradation 45, 46. As expected, ubiquitinated CHOP (Ub-CHOP) levels were increased in hepatocytes with *Txnip* KD and decreased in hepatocytes with *Txnip* OE (Figure 5C,D). These results suggest that TXNIP inhibits ubiquitination of CHOP protein, thus leading to the accumulation of CHOP protein.

### TXNIP interacts with CHOP both *in vitro* and *in vivo*

**Figure 6:**
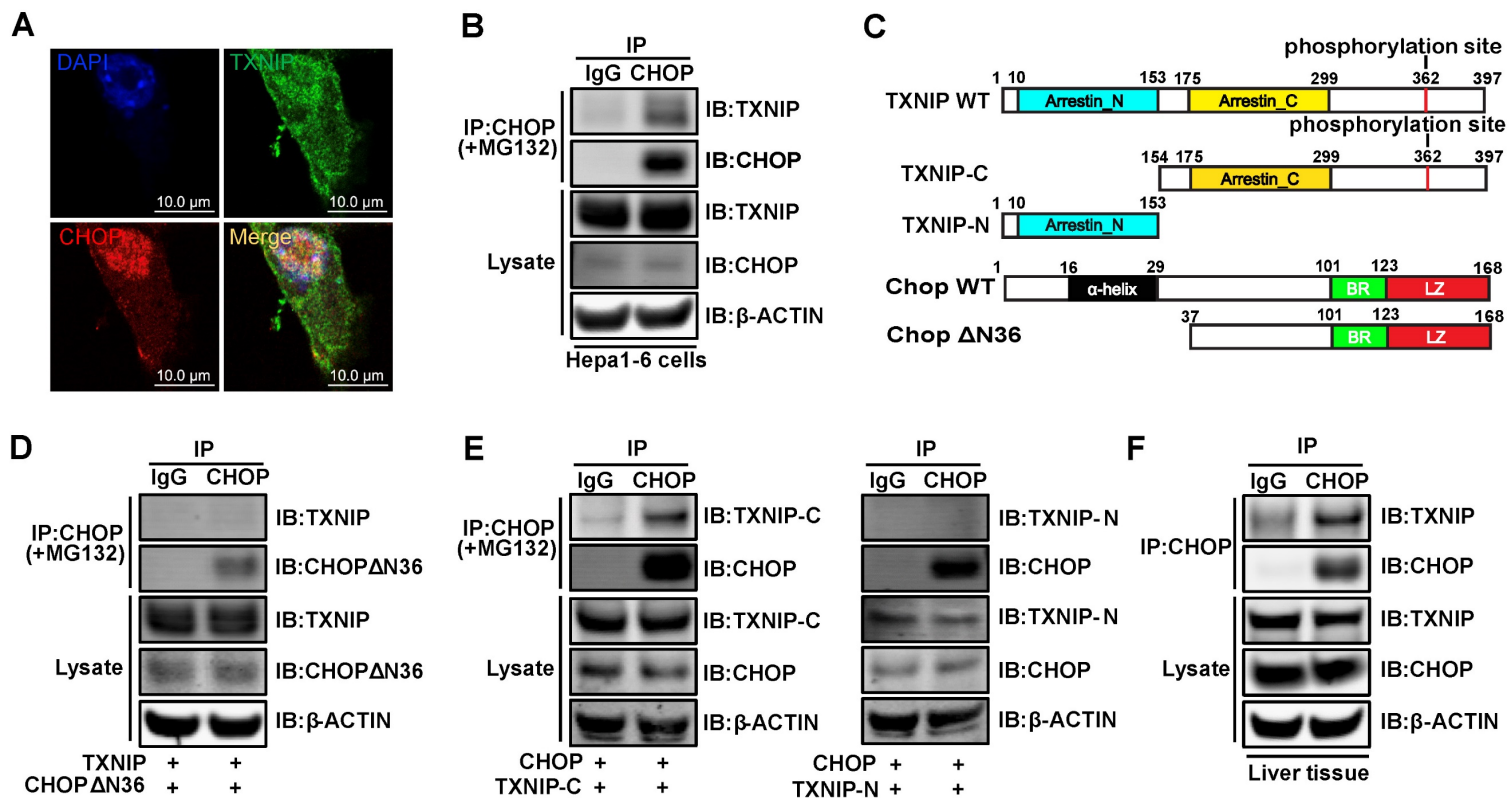
** TXNIP physically binds to CHOP *in vitro* and *in vivo*.** (A) TXNIP colocalized with CHOP. After Hepa1-6 cells were transiently transfected with *Txnip* and *Chop* overexpression vectors for 24 h, the cells were then incubated with 5μM MG132 for 4 hours, followed by an immunofluorescence experiment. Scale bars: 10 µm. (B) Physical interaction of TXNIP and CHOP. Same as in (A) but equal amounts of Hepa1-6 cell lysates were immunoprecipitated with negative control IgG or CHOP antibodies followed by western blot. (C) Schematic representation of WT TXNIP and its C/N-terminal truncations (upper panel), WT CHOP and its ΔN36 (α-helix domain deletion) truncation (lower panel). (D) The α-helix domain of CHOP is critical for TXNIP binding. Hepa1-6 cells were transfected with the WT TXNIP and CHOP ΔN36 truncation construct. Cell lysates were immunoprecipitated with CHOP antibody, followed by western blot with anti-TXNIP or anti-CHOP antibody. (E) The C-terminal end of TXNIP binds to CHOP. Hepa1-6 cells were transfected with the WT CHOP and TXNIP N-terminus or C-terminus constructs. Cell lysates were immunoprecipitated with CHOP antibody, and immunoblotted with anti-V5 (anti-TXNIP) or anti-CHOP antibody. (F) Immunoprecipitation analysis of interaction between the TXNIP and CHOP in GAN diet NASH mouse liver.

It has been shown previously that CHOP is degraded through the ubiquitin-proteasome pathway by directly binding to its regulators 45, 46. This led us to hypothesize that TXNIP may interact with CHOP directly to regulate CHOP protein stability. In Hepa1-6 cells, we detected colocalization of TXNIP and CHOP by confocal microscopy (Figure 6A). Additionally, CHOP protein co-immunoprecipitated with TXNIP protein in Hepa1-6 cells (Figure 6B). To better understand the molecular interaction between TXNIP and CHOP, we generated several truncated versions of the proteins (Figure 6C). Previous studies reported that the first 36 amino acids of CHOP are critical for CHOP degradation 45, 46. To determine whether the first 36 amino acids of CHOP are necessary for its binding to TXNIP, we constructed CHOPΔN36 (lacking α-helix domain) truncation for Co-IP assay (Figure 6C). We found that the deletion of these 36 amino acids of CHOP abrogated its ability to bind to TXNIP (Figure 6D). This result suggests that the N-terminal region of CHOP containing α-helix domain is necessary for the interaction. TXNIP contains two arrestin domains which are critical for protein binding 42. Therefore, we generated two truncation mutants of TXNIP, which contain the arrestin N (N-terminal) and arrestin C (C-terminal), respectively (Figure 6C). Co-immunoprecipitation experiments indicated that the C-terminal end but not the N-terminal end of TXNIP protein is necessary for its interaction with CHOP (Figure 6E). The interaction between TXNIP and CHOP was also confirmed in mouse liver (Figure 6F). Taken together, these data suggest that TXNIP protein interacts with CHOP protein both *in vitro* and *in vivo*.

### Selective KD of hepatic *Txnip* protects mice from NASH

**Figure 7:**
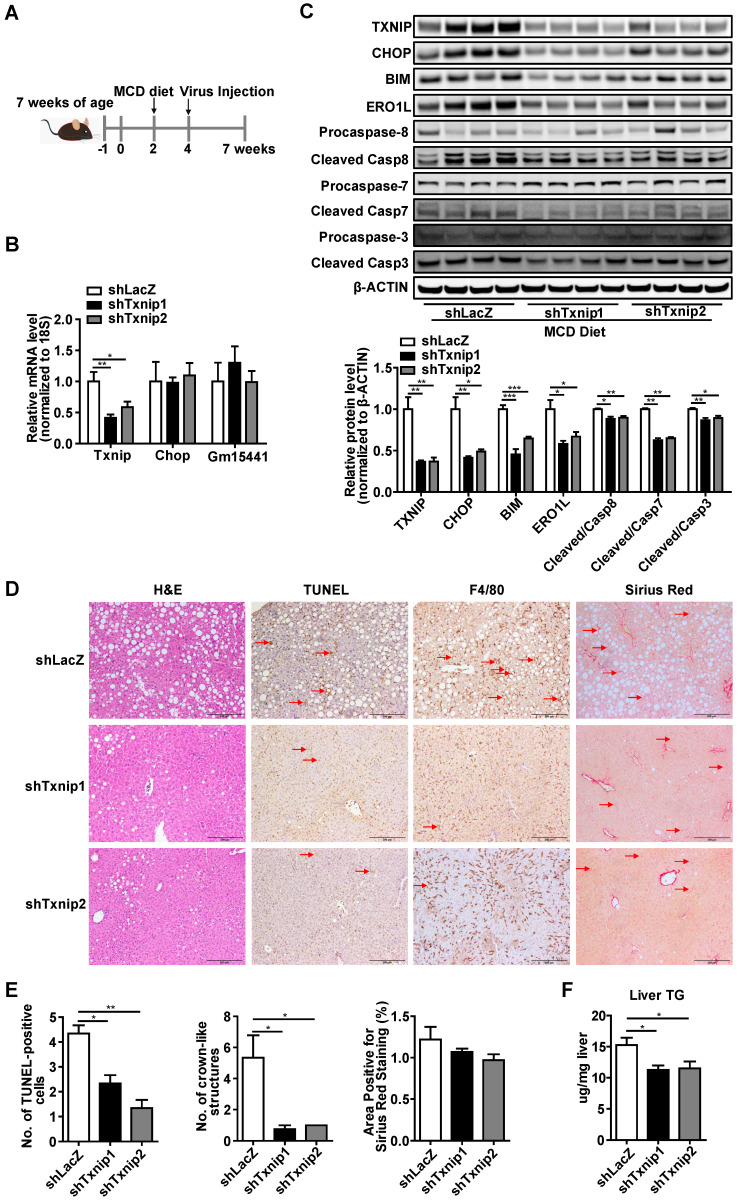
** Deficiency of *Txnip* attenuates NASH symptoms in MCD-NASH mice liver.** (A) Schematic diagram of experimental design in MCD-NASH model: 10 weeks old mice were fed with an MCD diet for 5 weeks. After 2 weeks of MCD diet feeding, mice were injected with shLacZ or shTxnip adenovirus along with continuous MCD diet feeding. (B) RT-PCR analysis of *Txnip, Chop,* and *Gm15441* levels in the indicated mice liver (n = 5-7). (C) Western blot analysis of TXNIP and CHOP levels as well as CHOP downstream targets in the indicated mouse livers. (D) H&E staining, terminal deoxynucleotidyl transferase-mediated dUTP nick end-labeling (TUNEL) staining, immunohistochemical staining for F4/80, and Sirius Red staining of the liver section. Scale bar: 200 µm. Red arrows indicate TUNEL-positive cells (in TUNEL staining image) or crown-like structures (in F4/80 staining image) or positive area (in Sirius Red staining image). (E) Quantification of various staining in (D) (n = 4). (F) Liver TG level in each group (n = 5-7). Data shown as mean ± SEM (*P < 0.05, **P < 0.01, ***P < 0.001 versus control group).

**Figure 8:**
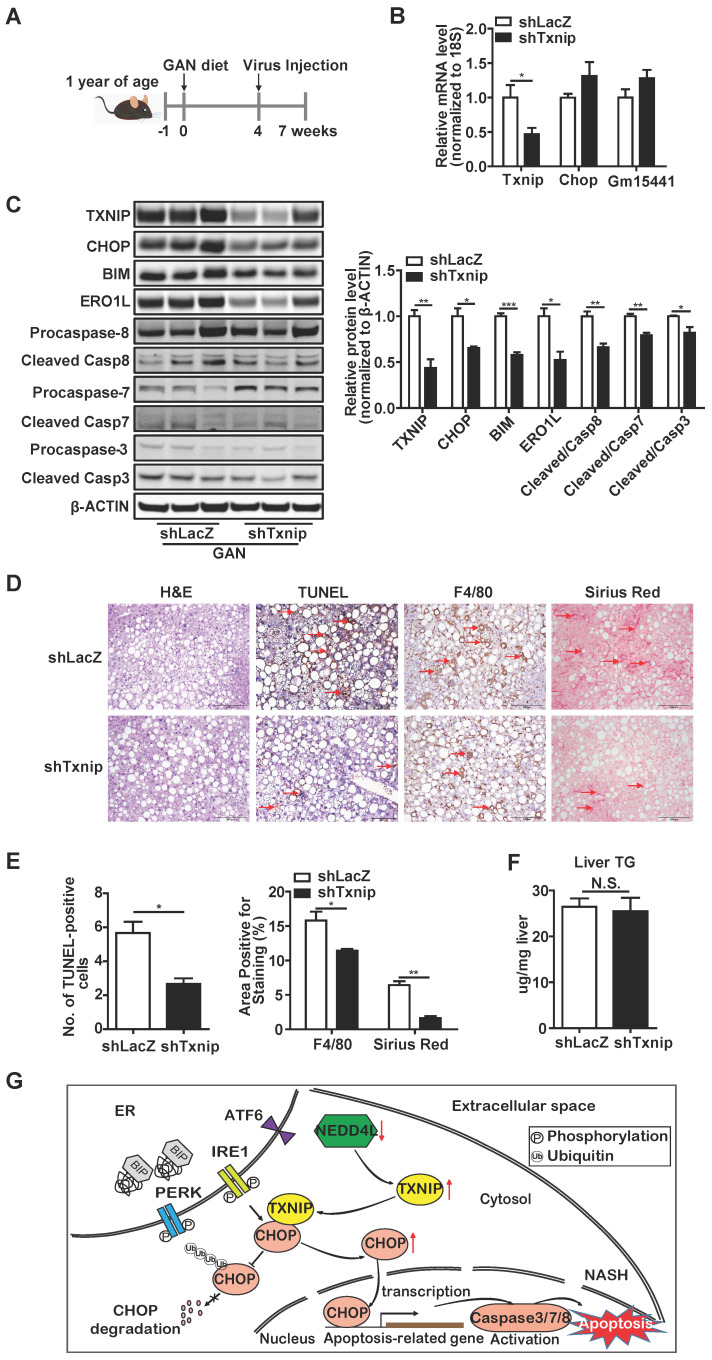
** Deficiency of *Txnip* attenuates NASH symptoms in age-associated NASH mice liver.** (A) Schematic diagram of experimental design in age-associated NASH model: one-year-old mice were fed with GAN diet for 7 weeks. After 4 weeks of GAN diet feeding, mice were injected with shLacZ or shTxnip adenovirus along with continuous GAN diet feeding. (B) RT-PCR analysis of *Txnip*, *Chop,* and *Gm15441* levels in the indicated mice liver (n = 5). (C) Western blot analysis of TXNIP and CHOP levels as well as CHOP downstream targets in the indicated mouse livers. (D) H&E staining, terminal deoxynucleotidyl transferase-mediated dUTP nick end-labeling (TUNEL) staining, immunohistochemical staining for F4/80, and Sirius Red staining of the liver section. Scale bar: 200 µm. Red arrows indicate TUNEL-positive cells (in TUNEL staining image) or crown-like structures (in F4/80 staining image) or positive area (in Sirius Red staining image). (E) Quantification of various staining in (D) (n = 4). (F) Liver TG level in each group (n = 5). (G) A schematic overview of the role of NEDD4L-TXNIP-CHOP axis during NASH development. In the context of NASH, deficiency of E3 ligase NEDD4L leads to TXNIP protein accumulation, which in turn binds to and promotes CHOP protein accumulation and activates apoptotic pathway. Data shown as mean ± SEM (*P < 0.05, **P < 0.01, ***P < 0.001 versus control group, N.S., not significant).

To investigate the gene- and liver- specific role of *Txnip* in NASH, we used adenovirus (that targets the liver) mediated *Txnip* shRNA (that does not affect the *Txnip* antisense lncRNA *Gm15441*). Considering that aging is a key factor for the development of NASH, we used both young mice MCD diet (Figure 7) and aged mice GAN diet (Figure 8) NASH models. Ten-week-old mice were fed an MCD diet for 5 weeks, and LacZ shRNA (shLacZ, control) or *Txnip* shRNA (shTxnip) adenovirus was injected 3 weeks before the end of the experiment (Figure 7A). As expected, both *Txnip* mRNA and protein expression were decreased without affecting *Gm15441* by shTxnip (Figure 7B, C) while *Chop* depletion was detectable only at the protein level in the liver (Figures 7B, C). Consistent with decreased CHOP protein, CHOP-specific downstream targets BIM and ERO1L as well as several caspase proteins in the apoptotic pathway were also significantly inhibited in the livers of MCD-fed mice with *Txnip* KD (Figure 7C), where decreased evidence of apoptosis was detected (Figure 7D, E). Immunohistochemical detection of the macrophage marker *F4/80* further revealed attenuation of macrophage infiltration upon *Txnip* KD (Figure 7D, E).

In addition, one-year-old mice were fed a GAN diet for 7 weeks in total, and LacZ shRNA (control) or *Txnip* shRNA adenovirus was injected 3 weeks before the end of the experiment (Figure 8A). In this model system, *Txnip* depletion was effective without affecting *Gm15441* (Figure 8B, C) and recapitulated results found in young mice with MCD-induced NASH with regard to effects on expression of CHOP and its downstream targets as well apoptosis and macrophage infiltration (Figure 8C-E). By contrast, while minimal hepatic fibrosis was observed in the young mice fed with MCD diet (Figure 7D, E), hepatic fibrosis was detected in aged mice and significantly reduced with *Txnip* KD (Figure 8D,E). Lastly, liver TG level was reduced in the *Txnip* KD group in the young mice with MCD-induced but not in age-associated NASH (Figure 7D,F and 8D,F). Additionally, we measured several key proteins in the autophagy pathway and found there were no changes (Supplementary Fig. S3). Besides, we measured the expression of *Txnip* in other tissues (lung and spleen) and found its expression was not changed by shTxnip adenovirus, supporting adenovirus mainly targeting the liver (Supplementary Fig. S4). Taken together, these data indicate that knockdown of *Txnip* protects mice from NASH in both young and aged NASH mouse models.

To support that TXNIP functions through CHOP, we rescued CHOP expression in Txnip knockdown NASH mouse liver. We found that due to the rescued CHOP protein levels, the downregulation of apoptotic (ERO1L), inflammation (*F4/80*), and fibrosis (*Col1a1* and *Col3a1*) genes by *Txnip* knockdown was reversed (Supplementary Fig. S5). These results suggest that CHOP is involved in the reduced hepatic apoptosis, inflammation, and fibrosis mediated by Txnip knockdown.

Adenovirus targets not only hepatocytes but also non-parenchymal cells (NPCs) (Supplementary Fig. S6A). To determine whether NEDD4L-TXNIP-CHOP axis drives NASH development in hepatocytes *in vivo*, we first measured the expression levels of TXNIP, CHOP, and NEDD4L in hepatocytes and NPCs from the livers of mice fed either a normal diet or an MCD diet. We found that the accumulation of TXNIP in the liver was mainly contributed by the accumulation of TXNIP in hepatocytes of the MCD diet fed mouse liver (Supplementary Fig. S6B). We also found that CHOP was increased while NEDD4L was decreased in hepatocytes as we observed in the total liver samples. Besides, TXNIP was mainly expressed in hepatocytes in the MCD diet fed mouse liver (Supplementary Fig. S6B). Next, we knocked down *Txnip* in hepatocytes using GalNAc conjugated siRNA (targets hepatocytes specifically) 47, and found that the GalNAc *Txnip* siRNA results were similar to the *Txnip* shRNA adenovirus results (Supplementary Fig. S7). These results suggest that the NEDD4L-TXNIP-CHOP axis mainly drives NASH development in hepatocytes.

## Discussion

NAFLD is a multifactorial metabolic disorder that has emerged as the most common chronic liver disease worldwide 48. NASH is a severe form of NAFLD that requires treatment, but no FDA-approved therapy is yet available. Untreated NASH can lead to liver failure or cancer, and NASH has become the second leading indication for liver transplantation in the USA 49. Improved understanding of the molecular pathogenesis of NASH development is critical for developing novel therapies to treat NASH. In this study, we defined the pathogenic role of hepatic TXNIP in NASH and identified a novel NEDD4L-TXNIP-CHOP axis that is critical for the pathogenesis of NASH (Figure 8G). As our study was conducted using mouse models, it can serve as a basis and an inspiration for future human research in this area. TXNIP has already been tested in clinical trials as a therapeutic target for diabetes, suggesting that targeting TXNIP to treat NASH is feasible. The current study is a significant step toward designing clinical trials for patients with NASH by targeting the NEDD4L-TXNIP-CHOP axis.

Although TXNIP protein is increased in NAFL and further increased in NASH in livers of human patients 24, expression of *Txnip* in NASH mouse models is not well defined. We report that TXNIP protein accumulates in the livers of four different NASH mouse models, including one novel age-associated NASH mouse model (Figure 1A-C and Figure 2M), which is consistent with findings in liver samples derived from patients with NASH. Considering the varied mechanisms of different NASH mouse models, the shared elevation of TXNIP protein in multiple NASH mouse models robustly support the critical role of TXNIP in NASH development. In addition, the underlying molecular mechanism by which TXNIP protein accumulates in NASH is unclear. Here, we found that upregulation of TXNIP protein is not due to an increase in mRNA levels in NASH mouse livers (Figure 1A-C, Figure 2M, and Figure 3A), suggesting a post-transcriptional regulation. Further investigation identified the impaired ubiquitination of TXNIP as a novel mechanism that leads to TXNIP protein accumulation in NASH mouse liver (Figure 3C). The specificity of protein ubiquitination is mediated by the E3 ubiquitin ligases, which can transfer ubiquitin from E2 ubiquitin-conjugating enzymes to substrates 42. To take a step further, we identified NEDD4L as a novel E3 ubiquitin ligase for the ubiquitination on TXNIP protein (Figure 3). These findings improved our understanding of the molecular mechanisms underlying hepatic TXNIP protein accumulation under NASH condition. TXNIP-mediated oxidative stress and CHOP-mediated ER stress are two important and cross-talked cell stress pathways that can promote cell death in NASH 50. However, the underlying molecular mechanism of their crosstalk is not fully understood. In this study, we also reported a novel mechanism of crosstalk between oxidative stress and ER stress in NASH that is mediated by the interaction between TXNIP and CHOP protein. There may be additional downstream targets of TXNIP in NASH development, an RNA-seq analysis will be included in our future study to identify new targets. Taken together, the novel NEDD4L-TXNIP-CHOP axis identified in this report could offer new opportunities for developing novel therapy against NASH.

Both pathogenic and protective roles for TXNIP in NASH mouse models have been reported 30-32, 51. For example, Min-Jung Park *et al*. reported that TXNIP mediates HFD-hepatic lipogenesis and inflammation, which showed that TXNIP plays a pathogenesis role in NAFLD. In their study, they used the *Txnip* whole body KO mice, which deleted the sequence from exon one to exon eight of *Txnip* 51. Islam N Mohamed *et al*. also reported that *Txnip* deletion ameliorates HFD-induced steatosis, inflammatory and fibrotic response, suggesting a pathogenic role of TXNIP in NASH 30. In their study, they used *Txnip* whole body KO mice which lost the exon two to exon eight of *Txnip*. Md Kaimul Ahsan *et al*. also reported that TXNIP (alternative name: TPB-2) plays a pathogenic role in MCD diet induced NASH mouse model, and inhibitors of TXNIP can be used for the prevention or treatment of NASH 31. In their study, the author used the TBP-2 whole body KO mice which lost the exon one to exon four of *Txnip*. On the contrary, Hee-Seon Park *et al*. elaborated a protective role of TXNIP in MCD diet induced NASH 32. They found that elevated TXNIP could ameliorates steatohepatitis by interacting with PRKAA and thereby inducing autophagy and FAO. In their study, the author used the *Txnip* whole body KO mice, which lost the exon one to exon eight. Therefore, such a discrepancy may be partly attributable to the deletion of different *Txnip* exons and different diets. Moreover, the phenotype of the whole-body *Txnip* KO mice under NASH conditions might also be impacted by the simultaneous deletion of *Txnip* antisense lncRNA Gm15441 at the *Txnip* locus. Studies have shown that *Gm15441* regulates liver inflammation by affecting NLR family pyrin domain containing 3 (NLRP3) inflammasome activation, caspase-1 (CASP1) cleavage, and pro-inflammatory interleukin 1β (IL1B) maturation 34. To selectively knock down *Txnip* in a liver-specific manner, we designed shRNA targeting *Txnip*-specific mRNA sequence not its *Gm15441* RNA overlapping sequence, and utilized adenovirus-mediated gene therapy to target this shRNA to the liver. We found that *Txnip*-specific KD in the liver significantly alleviated NASH in both young and aged NASH mice (Figure 7 and 8). We also found that GalNAc siRNA mediated hepatocyte-specific *Txnip* KD alleviated NASH (Supplementary Fig. S7). However, in contrast to *Txnip* whole body KO mice 32, liver- and gene-specific *Txnip* KD failed to regulate autophagy pathway genes (Supplementary Fig. S3), indicating the potential involvement of other tissues and Gm15441. Taken together, these important findings strongly support the pathogenic role of *Txnip* in NASH. In addition, the results from this study demonstrate *Txnip*-specific function in NASH mouse liver without the interference of *Gm15441*, which is critical for better understanding the role of *Txnip* in NASH. As TXNIP has been considered a promising therapeutic target for diabetes 52, targeting TXNIP may be an ideal therapeutic strategy for patients with both NASH and diabetes.

In conclusion, this study more precisely defined the role of TXNIP protein in NASH, including in a novel age-associated NASH mouse model and identified a novel NEDD4L-TXNIP-CHOP pathway and TXNIP-CHOP inter-molecular interactions. This study demonstrated that TXNIP and CHOP proteins form a complex to regulate NASH development. This study also demonstrated that impaired TXNIP protein degradation is a key factor for the TXNIP protein accumulation in NASH mouse livers. Besides, this study provides novel insights into the regulation of *Txnip* at post-translational levels (E3 ligase mediated protein degradation) under NASH conditions. Overall, this study improves our understanding of the liver- and gene-specific role of *Txnip* in NASH development and will significantly accelerate the development of novel therapeutic strategies that targeting NEDD4L-TXNIP-CHOP axis to treat NASH.

## Supplementary Material

Supplementary figures and tables.

## Abbreviations

AMPK: AMP-dependent protein kinase
ALT: alanine aminotransferase
*Chop*: CCAAT-enhancer-binding protein homologous protein
CDS: coding sequence
CHX: cycloheximide
Co-IP: co-immunoprecipitation
ER: endoplasmic reticulum
KO: knockout
KD: knockdown
MCD: Methionine and Choline Deficient
NASH: nonalcoholic steatohepatitis
*Nedd4l*: Nedd4 like E3 ubiquitin protein ligase
NAFLD: non-alcoholic fatty liver disease
NAFL: non-alcoholic fatty liver
OE: overexpression
PPXY: proline proline X tyrosine
ROS: reactive oxygen species
shRNA: short hairpin RNA
siRNA: small-interfering RNA
*Txnip*: thioredoxin-interacting protein
TXN: thioredoxin
TUNEL: terminal deoxynucleotidyl transferase-mediated dUTP nick end-labeling
TG: triglyceride
TC: total cholesterol
Ub-CHOP: ubiquitinated CHOP
VDUP1: Vitamin D up-regulated protein 1
WAT: white adipose tissue
