# Supplementary material for: A novel NEDD4L-TXNIP-CHOP axis in the pathogenesis of nonalcoholic steatohepatitis
Source: Theranostics. 2023 Apr 9;13(7):2210–25. doi: 10.7150/thno.81192 (PMC10157740; doi:10.7150/thno.81192)
Supplement: Supplementary file 1 — Supplementary figures and tables. [file thnov13p2210s1.pdf]

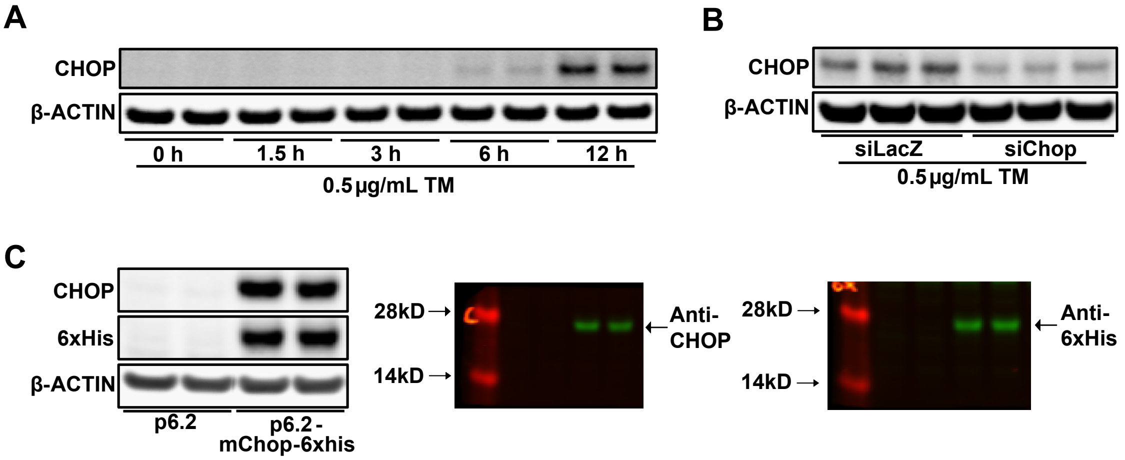

Supplementary Fig. S1. Validation of CHOP antibody. (A) Western Blot analysis of CHOP in Hepa1-6 cells treated with 0.5 μg/mL Tunicamycin (TM) for different times. (B) Western Blot analysis of CHOP in Hepa1-6 cells exposed to 0.5 μg/mL Tunicamycin (TM) for 6 hours after transfection with *Chop* siRNA or control siRNA for 24 hours. (C) Western Blot analysis of CHOP in Hepa1-6 cells transfected with *Chop* overexpression or control vector for 36 hours. The location of CHOP protein detected by CHOP antibody (middle) is the same as that detected by 6xhis antibody (right).

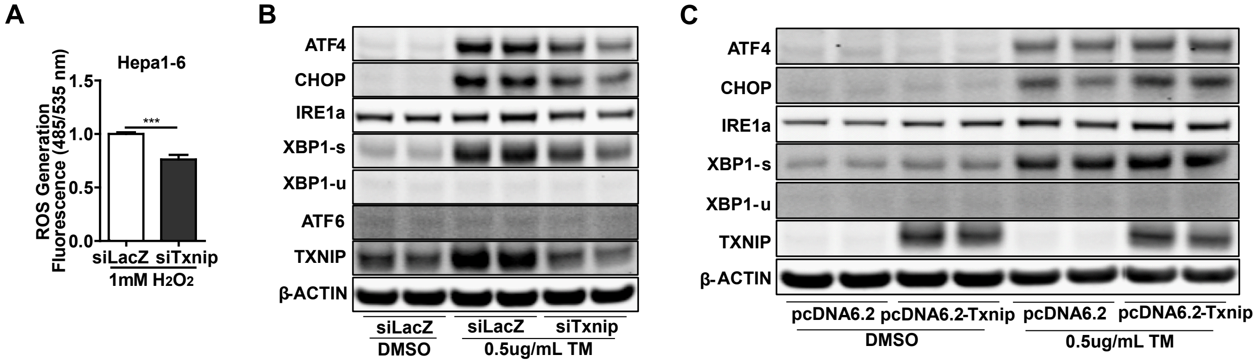

Supplementary Fig. S2. *Txnip* regulates oxidative stress and ER stress in vitro. (A) ROS levels in Hepa1-6 cells transfected with siTxnip for 48 hours followed by 1mM H2O2 treatment for 4 hours (n=7). Data shown as mean ± SEM (***P<0.001 versus control group). (B) Western blot analysis of ER stress pathway in Hepa1-6 cells exposed to 0.5 μg/mL Tunicamycin (TM) for 6 hours after transfection with *Txnip* siRNA or control siRNA for 24 hours. (C) Western blot analysis of ER stress pathway in Hepa1-6 cells exposed to 0.5 μg /mL Tunicamycin (TM) for 6 hours after transfection with *Txnip* overexpression vector or control vector for 24 hours.

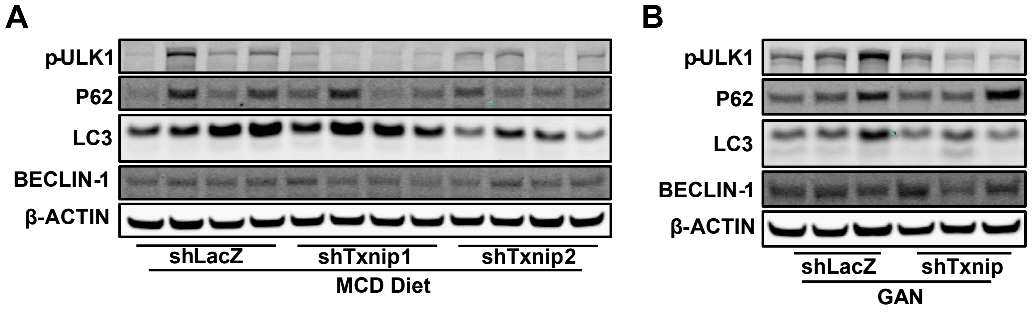

Supplementary Fig. S3. Autophagy pathway is not affected by *Txnip* in NASH models. (A) Western blot analysis of autophagy pathway in MCD-induced NASH mouse livers with or without *Txnip* knockdown (10 weeks old mice were fed with an MCD diet for 5 weeks. After 2 weeks of MCD diet feeding, mice were injected with shLacZ or shTxnip adenovirus along with continuous MCD diet feeding, shown in Figure 7). (B) Western blot analysis of autophagy pathway in age-associated NASH mouse livers with or without *Txnip* knockdown (one-year-old mice were fed with GAN diet for 7 weeks. After 4 weeks of GAN diet feeding, mice were injected with shLacZ or shTxnip adenovirus along with continuous GAN diet feeding, shown in Figure 8).

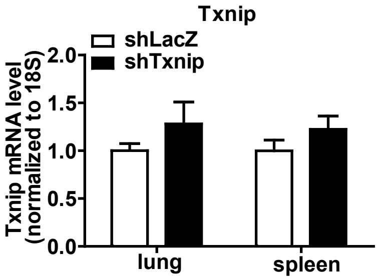

Supplementary Fig. S4. Adenovirus shTxnip does not affect the *Txnip* expression in the lung and spleen. RT-PCR analysis of *Txnip* mRNA levels in the lung and spleen of age-associated NASH mice with or without *Txnip* knockdown (n=8). Data shown as mean ± SEM.

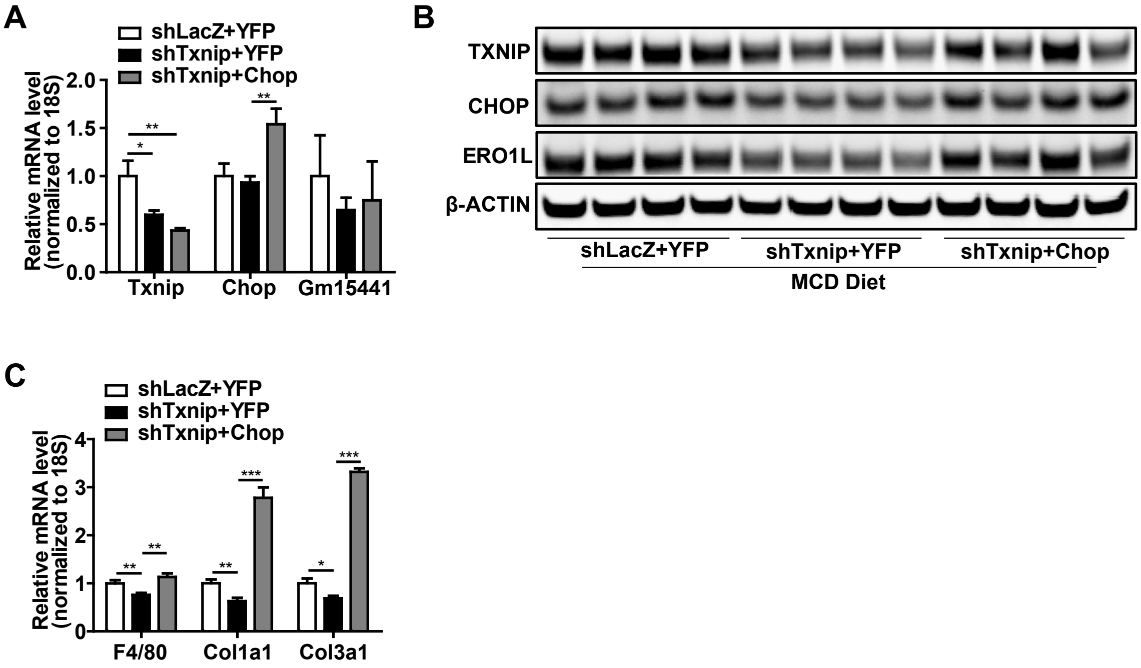

Supplementary Fig. S5. Overexpression of *Chop* blocks the effect of *Txnip* knockdown on inflammation and fibrosis in MCD-NASH model. (A) RT-PCR analysis of *Txnip*, *Chop,* and *Gm15441* levels in the indicated mouse livers (n=8). (B) Western blot analysis of TXNIP and CHOP levels as well as CHOP downstream target ERO1L in the indicated mouse livers. (C) RT-PCR analysis of *F4/80*, *Col1a1,* and *Col3a1* levels in the indicated mouse livers (n=8). Data shown as mean ± SEM (*P<0.05, **P<0.01, ***P<0.001).

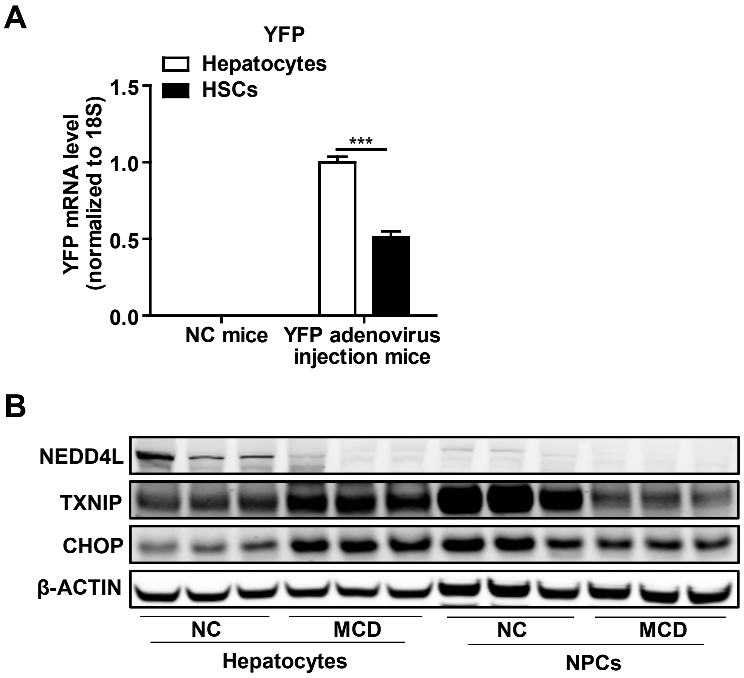

Supplementary Fig. S6. TXNIP is mainly elevated in the hepatocytes under NASH condition.

(A) RT-PCR analysis of YFP mRNA level in isolated hepatocytes and NPCs from mice injected with YFP overexpression adenovirus (n=3). (B) Western blot analysis of NEDD4L, TXNIP, and CHOP levels in isolated hepatocytes and NPCs. Data shown as mean ± SEM (***P<0.001).

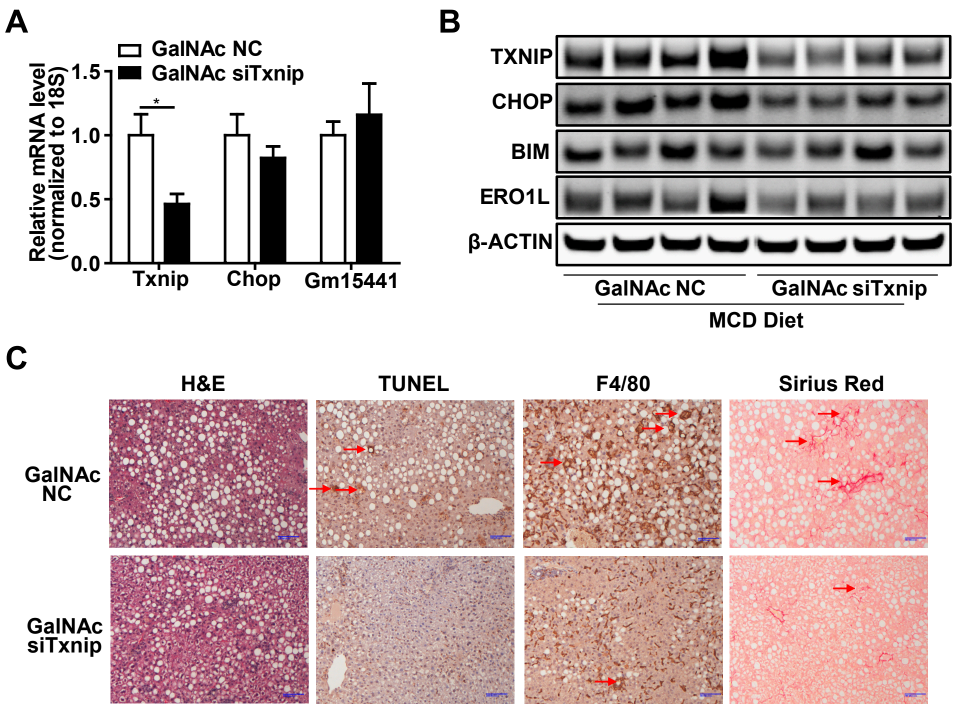

Supplementary Fig. S7. GalNAc siRNA mediated hepatic *Txnip* deficiency attenuates NASH symptoms in MCD induced NASH model. (A) RT-PCR analysis of *Txnip*, *Chop* and *Gm15441* levels in the indicated mice liver (n=6). Data shown as mean ± SEM (*P<0.05). (B) Western blot analysis of TXNIP and CHOP levels as well as CHOP downstream targets BIM and ERO1L in the indicated mouse livers. (C) H&E staining, terminal deoxynucleotidyl transferase-mediated dUTP nick end-labeling (TUNEL) staining, immunohistochemical staining for F4/80, and Sirius Red staining of the liver section. Scale bar: 200 μm. Red arrows indicate TUNEL-positive cells (in TUNEL staining image) or crown-like structures (in F4/80 staining image) or positive area (in Sirius Red staining image).

Supplementary Tables

Table S1. Quantitative real-time PCR primers.

Gene	Forward primer	Reverse primer
Ccl2	5’-GAAGGAATGGGTCCAGACAT-3’	5’-ACGGGTCAACTTCACATTCA-3’
F4/80	5’-CTTTGGCTATGGGCTTCCAGTC-3’	5’-GCAAGGAGGACAGAGTTTATCGTG-3’
Tgfb	5’-GTGGAAATCAACGGGATCAG-3’	5’-ACTTCCAACCCAGGTCCTTC-3’
Acta2	5’-TCCATCGTCCACCGCAAAT-3’	5’-GCCAGGGCTACAAGTTAAGG-3’
Timp1	5’-CTTGGTTCCCTGGCGTACTC-3’	5’-ACCTGATCCGTCCACAAACAG-3’
Col1α1	5’-GAGCAGACGGGAGTTTCTCCT-3’	5’-CATGTAGACTCTTTGCGGCTG-3’
Col1α2	5’-AGGATTGGTCAGAGCAGTGT-3’	5’-TCCACAACAGGTGTCAGGGT-3’
Col3α1	5’-CCACCCAATACAGGTCAAATGC-3’	5’-TGAGTATGACCGTTGCTCTGC-3’
Txnip	5’-CATGAGGCCTGGAAACAAAT-3’	5’-ACTGGTGCCATTAGGTCAGG-3’
Chop	5’-GGGAAACAGCGCATGAAGGA-3’	5’-GCGTGATGGTGCTGGGTACA-3’
Itch	5’-TGGGTAGTCTGACCATGAAATCT-3’	5’-GGGGTAACAATAACTGTGAGGG-3’
Nedd4	5’-TCGGAGGACGAGGTATGGG-3’	5’-GGTACGGATCAGCAGTGAACA-3’
Nedd4I	5’-GTCCGGCTGTTCCGTACTC-3’	5’-AGGCCATAGTAGGGGTTAAACAT-3’
Smurf1	5’-AGCATCAAGATCCGTCTGACA-3’	5’-CCAGAGCCGTCCACAACAAT-3’
Smurf2	5’-AAACAGTTGCTTGGGAAGTCA-3’	5’-TGCTCAACACAGAAGGTATGGT-3’
Wwp1	5’-TAAAGGTAACGGTTTCTAGTGCC-3’	5’-TGTGGGGTCACATTTACAATCAG-3’
Wwp2	5’-TTTGAGAAGTCCCAGCTTACCC-3’	5’-CTCCAGACCTTCAGATCCAAATG-3’
Hecw2	5’-AGAGCCCACACTTGTTTTAACC-3’	5’-TCCAAAGGTACTGGTCTCTTCAA-3’
18S	5’-AGTCCCTGCCCTTTGTACACA-3’	5’-CGATCCGAGGGCCTCACTA-3’

Table S2. siRNA interference sequences

Target	Sequence (5’-3’)
si-Txnip-1	GGAUGUUGGAAAUGAAGAA
si-Txnip-2	GGGUGACAUUCUACAUUGA
si-Nedd4I-1	GCAGAAAUACGACUACUUU
si-Nedd4I-2	GGUCCUCAGCUGUUUACAA
si-Chop	GAUUCCAGUCAGAGUUCUA

Table S3. shRNAs sequences

Target	Sequence (5'-3')
Txnip sh1F	CACCG GGATGTTGGAAATGAAGAA TTCAAGAGA TTCTTCATTTCCAACATCC
Txnip sh1R	AAAA GGATGTTGGAAATGAAGAA TCTCTTGAA TTCTTCATTTCCAACATCC C
Txnip sh2F	CACCG GGGTGACATTCTACATTGA TTCAAGAGA TCAATGTAGAATGTCACCC
Txnip sh2R	AAAA GGGTGACATTCTACATTGA TCTCTTGAA TCAATGTAGAATGTCACCC C
